# Assessment of nutritional quality of *taro* (*Colocasia esculenta* L. Schott.) genotypes of the Eastern Himalaya, India

**DOI:** 10.3389/fnut.2025.1567829

**Published:** 2025-04-09

**Authors:** Hammylliende Talang, Gabriella T. Mawlong, Lanamika Kjam, M. Bilashini Devi, Bishal Gurung, Niraj Biswakarma, Nongmaithem Uttam Singh, Veerendra Kumar Verma, Heiplanmi Rymbai, Palavalasa Raviteja, Bapi Das, Thejangulie Angami, Aabon W. Yanthan, Sandip Patra, Badapamain Makdoh, Rumki H. Ch Sangma, Shiwot Ruth Assumi, Christy B. K. Sangma, L. Joymati Chanu, Samarendra Hazarika

**Affiliations:** ^1^ICAR Research Complex for NEH Region, Umiam, Meghalaya, India; ^2^North Eastern Hill University, Umshing Mawkynroh, Shillong, Meghalaya, India; ^3^ICAR Research Complex for NEH Region, Tripura Centre, Lembucherra, Tripura, India; ^4^ICAR Research Complex for NEH Region, Arunachal Pradesh Centre, Basar, Arunachal Pradesh, India; ^5^ICAR Research Complex for NEH Region, Nagaland Centre, Medziphema, Nagaland, India

**Keywords:** taro, genotypes, bio-chemical, antioxidant, principal component analysis

## Abstract

**Introduction:**

The eastern Himalayan region of India with diverse agro-climatic conditions is one of the important hotspots of the world’s biodiversity. A wide range of genetic variability of plant species like Colocasia is available in the region which is consumed by the local tribes.

**Materials and methods:**

A field study was conducted during 2022–23 to evaluate the yield, biochemical, mineral, and an-tioxidant parameters of 30 *Colocasia esculenta* L. Schott. genotypes under a split-plot design with three replications.

**Results and discussion:**

Significant (*p* < 0.05) variations were observed among genotypes for all traits. Tamachongkham exhibited the highest corm weight and yield, while Tamitin recorded the maximum cormel weight and total yield. Megha Taro-2 and Megha Taro-1 had the highest cormel numbers and cormel yield, respectively. In mineral composition, Tamitin had the highest N, K, Zn, Cu, and Mn, Tagitung White recorded the highest P, and BCC-2 had the highest Fe and Ca + Mg. Biochemically, Tamachongkham had the highest dry matter content; Khweng-2 had the highest starch, total sugar, and reducing sugar; Rengama had the highest crude protein, and crude fiber; and Mairang Local had the highest ash content. A significant positive correlation was observed between total yield and corm, cormel yield, cormel weight, and corm weight, while correlations with starch and other parameters were non-significant. Total phenolic content and anthocyanin were significantly correlated with Ferric Reducing Antioxidant Power (FRAP). Genotype-by-trait biplot analysis using the first two principal components (PC1: 19.4%, PC2: 14%) high-lighted total sugar, reducing sugar, cormel numbers, crude fiber, anthocyanin, and FRAP as major contributors to phenotypic diversity. The observed variations indicate the potential of these genotypes for future breeding programs aimed at improving taro production in the Eastern Himalayas.

## 1 Introduction

The Eastern Himalayan region of India with diverse agro-climatic conditions is one of the important hotspots of the world’s biodiversity. A wide range of genetic variability of plant species like Colocasia is available in the region which is consumed by the local tribes. Taro (*Colocasia esculenta* L. Schott.) also referred to as arvi or taro, belongs to the family Araceae comprising of 110 genera and more than 2000 species (1). It is cultivated in an area of around 1.9 million hectares around the world with an annual production of 12.39 MT (2). This crop is an integral part of dietary system of tribals and is grown abundantly in their jhum land or kitchen garden as mixed cropping. The most significant traits of taro are its adaptability, and capacity to give rise to high yields under a variety of conditions, particularly in tropical regions (3).

The underground corms and cormels, the primary edible components of taro, are highly nutritious and contain various bioactive compounds with potential health benefits (4). Taro is predominantly cultivated for its underground corms, which consist of 70–80% starch (5). It has been suggested that taro was traditionally grown to bridge seasonal food gaps, as it can yield well even under conditions where other crops may struggle due to various production constraints (5, 6). Morphologically, taro is an erect, monocotyledonous, and herbaceous perennial root crop. The plants possess a variety of chemical compounds that add to their therapeutic value, including alkaloids, glycosides, resins, volatile oils, gums, and tannins. Its tuber contains low fat with high protein and carbohydrate content, similar to other root crops. It is also a good source of potassium and has a moderate quantity of phosphorus. Besides, the tuber is also a richer source of vitamin B-complex than whole milk (7). In addition to being a very rich source of vitamin C, niacin, potassium, copper, and manganese, it is also a good source of iron, zinc, thiamine, riboflavin, and other minerals (8). The tuber has excellent digestibility. Its efficiency of effective simultaneous release of nutrients during digestion and absorption is attributed to the small granule size of its starch constituting the tuber (9). Despite the rich genetic diversity of taro (*Colocasia esculenta* L. Schott.) in the Eastern Himalayan region and its significance as a staple food for tribal communities, limited systematic studies have been conducted to assess the nutritional composition and agronomic potential of its diverse genotypes. While previous research has primarily focused on agronomic performance or isolated biochemical traits, comprehensive evaluations integrating yield attributes, biochemical composition, mineral content, and antioxidant properties across multiple genotypes remain scarce. Additionally, most studies have not employed multivariate analyses to identify superior genotypes for targeted breeding and genetic improvement. Given the increasing demand for nutrient-rich, climate-resilient crops, a deeper understanding of genotype-specific variations in taro is essential for its effective utilization in breeding programs. This study addresses this gap by evaluating 30 genotypes for key nutritional and antioxidant parameters, providing valuable insights for future genetic improvement and conservation efforts in the Eastern Himalaya region.

## 2 Materials and methods

### 2.1 Materials and experimental site

The study on the 30 genotypes of *Colocasia esculenta* L. cultivated at the Horticulture Experimental Farm of ICAR-Research Complex for North-Eastern Hill Region, Umiam, Meghalaya was performed during 2022–23. These 30 genotypes of *Colocasia esculenta* L. were selected based on their genetic diversity and wide representation from different geographical locations across the Eastern Himalayan region. This ensured a comprehensive evaluation of yield attributes, biochemical composition, and antioxidant properties for potential breeding and conservation efforts. The selected genotypes were treated as individual treatments. Each treatment was replicated thrice in split plot design. Throughout the trial, all the recommended cultivation techniques were adhered to in order to ensure optimal growth and yield. The genotypes were assigned to the blocks in the field experiment which was set up using split plot design. The split-plot design was chosen to efficiently evaluate the effects of multiple factors on the yield, biochemical, and antioxidant parameters of *Colocasia esculenta* L. genotypes. This design allowed for better control of variation by assigning genotypes to main plots and subplots, thus improving the precision of comparisons while accommodating field heterogeneity.

### 2.2 Quantitative analysis

Twenty-five matured corms and cormels of each genotype were used for carrying out all the analysis. The harvested samples were washed with distilled water and kept at room temperature for 10 min to remove the adhering water before analysis. The parameters, viz., corm and cormels weights (gm) were determined using an electronic balance (Adair Dutt-1620C). Corm yield (t ha^−1^), cormel yield (t ha^−1^) and total yield (t ha^−1^) of the genotypes were determined following standard methods (10, 11).

### 2.3 Determination of tissue nutrient

The leaf samples were collected and carefully washed to remove any surface contamination according to the method of Chapman (12). The samples were then oven dried and ground using a Wiley grinding machine to obtain a homogenous sample, which was subsequently digested in a tri-acid mixture of HClO_4_:HNO_3_:H_2_SO_4_ in a 2:5:1 ratio as described by Chapman and Pratt (13). The tri-acid extracts were used for the determination of total P as per the vanadomolybdate method given by Hesse (14), total K using the flame photometry method (13), micronutrients viz., copper, iron, manganese and zinc content using an atomic absorption spectrophotometer as described by Zasoski and Burau (15), and calcium and magnesium using the versenate (EDTA) method as described by Cheng and Bray (16). Total N was estimated following the Microkjeldahl method by Jackson (17).

### 2.4 Determination of biochemical attributes

Starch was estimated as per the procedure described by Hedge and Hofreiter (18), total sugar (Dubois et al. 1956) (19), Reducing Sugar (Miller 1959) (20), Oxalate (AOAC 1984) (21), crude protein (AOAC 1990) (22), crude fiber content (Maynard 1970) (23), ash content (James 1995) (24) with slight modification where two grams of the powdered sample was weighed (W_1_) into a pre-weighed empty crucible (W_0_) and placed into a muffle furnace until the sample was completely ashed at temperature 600°C. Dry-matter content of the samples was determined by oven-drying 100 g of freshly sliced tubers at 60°C, till a constant weight was attained and calculated as:


$$\mathrm{Dry}\;\mathrm{Matter}\;\left(\%\right)=\left(\mathrm{Dry}\;\mathrm{weight}/\mathrm{Fresh weight}\right)\times 100$$

### 2.5 Determination of antioxidant activity and anthocyanin

#### 2.5.1 Total phenolic content

The total phenolic content (TPC) was determined spectrophotometrically using the Folin–Ciocalteu method, as described by Keskin-Sasic et al. (25). The assay utilized Folin–Ciocalteu reagent, sodium carbonate (7.5% w/v), and gallic acid as the standard. For the analysis, 1 mL of the diluted sample extract was added to 2 mL of a 1:10 diluted Folin–Ciocalteu reagent and allowed to react for 10 min. Following this, 1.6 mL of sodium carbonate solution was added, and the mixture was incubated at room temperature for 30 min. The absorbance was then measured at 743 nm and TPC was expressed as gallic acid equivalents (GAE) in mg/100 g. The concentration of polyphenols in the samples was calculated using a standard calibration curve of gallic acid (y = 0.0232x–0.0602, R^2^ = 0.9885).


$$TPC\;\left(\mathrm{mg GAE}/100\;g\;dw\right)=\frac{\begin{array}{l} \mathrm{Conc. of GA from standard curve}\times \\ \mathrm{volume of extract}\times 100 \end{array}}{\mathrm{Weight of the sample}}$$

#### 2.5.2 Ferric reducing antioxidant power assay

The reducing power of the extracts was assessed as per the method described by Benzie and Strain (26). 0.1 mL of extracts/standard were taken in the labeled test tubes and 3 mL of FRAP Reagent was added to all the test tubes and the samples were allowed to react with the FRAP solution in the dark for 30 min followed by absorbance measurement. The FRAP values are expressed as millimoles of FeSO_4_ equivalents (FeSO_4_E) per 100 g of the sample using the standard curve constructed for different concentrations of Ferrous sulfate.


$$\mathrm{FRAP value}\;\left({\mathrm{mM FeSO}}_{4}E/100\;\mathrm{g dw}\right)=\frac{\begin{array}{l} \mathrm{Conc}{\mathrm{.of FeSO}}_{4}\;\mathrm{from}\\ s\tan\mathrm{dard curve}\times \\ \mathrm{volume of extract} \end{array}}{\mathrm{Weight of the sample}}$$

#### 2.5.3 DPPH (2,2-Diphenyl-1-picrylhydrazyl) radical scavenging assay

Free radical scavenging ability of the extracts was tested by DPPH radical scavenging assay as described by Shen et al. (27). For each sample/standard, 0.2, 0.4, 0.6, 0.8 and 1.0 mL were taken and the volume was made up to 1 mL with methanol followed by the addition of 3 mL DPPH solution. The samples were incubated for 30 min in the dark. Control solution was prepared by mixing methanol with DPPH solution. The absorbance was measured spectrophotometrically at 517 nm using methanol as blank. Percentage DPPH radical scavenging activity was calculated by the following equation:


$$\mathrm{DPPH Scavenging activity}\left(\%\right)=\frac{Ac-At}{Ac}\times 100$$

Where, Ac is the absorbance of the control reaction and At is the absorbance of the sample of the extracts. The antioxidant activity of the extract was expressed as IC50 (the concentration of sample required to decrease the absorption at 517 nm by 50%). The IC50 value was expressed as the concentration in milligram of extract per ml that inhibited the formation of DPPH radicals by 50%.

#### 2.5.4 Anthocyanin estimation

This estimation was carried out according to the procedure given by Srivastava and Kumar (28). A 10 g dried sample was mixed with 10 mL of ethanolic HCl and transferred to a 100 mL volumetric flask and the volume was made up to the mark with ethanolic HCl. The sample was kept overnight in the refrigerator at 4°C, filtered through Whatman no.1 filter paper and the OD was recorded at 535 nm.

Calculations:


$$\begin{array}{l} \mathrm{Total}\;OD/100\;g=\left(OD\times \mathrm{Volume made}\;\mathrm{up}\times 100\right)/\\ \mathrm{weight of sample taken} \end{array}$$

$$\mathrm{Anthocyanin}\;\left(\mathrm{mg}/100\;g\;dw\right)=\left(\mathrm{Total}\;OD/100\;g\right)/98.2$$

### 2.6 Statistical analysis

All tests were conducted in triplicates and the replicated data were statistically analyzed using IBM SPSS version 22.0. The results were expressed as mean ± standard deviation (SD) and subjected to one-way ANOVA followed by Tukey’s HSD (honestly significant difference) test at *p* < 0.05 for multiple comparisons. The relationships between yield, biochemical parameters, and antioxidant properties were evaluated using Pearson’s correlation coefficient. Pearson’s correlation analysis was conducted to evaluate the strength and direction of relationships among the examined traits, with Pearson’s correlation analysis was performed to assess the strength and direction of associations among the studied parameters, with statistical significance assessed at a 95% confidence level (*p* < 0.05). Furthermore, Principal Component Analysis (PCA) was employed to identify key variables contributing to the total variation and to reduce the dimensionality of the dataset. Eigenvalues greater than 1 were retained for interpretation, and factor loadings were analyzed to determine the contribution of each parameter to the principal components. A scree plot was used to visualize the variance explained by each component, providing insights into the underlying structure of the data.

Additionally, biplots were generated to illustrate the relationships between variables and principal components, aiding in the interpretation of trait clustering. The correlation matrix was examined to identify strong associations between parameters, which could further support PCA findings. Kaiser-Meyer-Olkin (KMO) and Bartlett’s test were performed to assess the suitability of the dataset for PCA. Higher KMO values indicated data adequacy, while Bartlett’s test ensured that correlations were sufficiently large for meaningful PCA. The cumulative variance explained by retained principal components was considered to determine the proportion of total variability captured. Finally, variables with high loadings in the same principal component were grouped, allowing for an integrated understanding of trait interactions and their overall contribution to data variability.

## 3 Results and discussion

### 3.1 Yield parameters

There was significant (*p* < 0.05) variation observed in yield and related parameters under study (Table 1). Among all the genotypes studied, the corm weight was recorded to be in the range of 80 ± 16.09 g to 641.67 ± 18.77 g where genotype Tamachongkham showed maximum value and the minimum in ML-3. Cormel weight was recorded highest in genotype Tamitin (151.67 ± 12.58 g) while Muktakeshi genotype recorded lowest cormel weight (19.53 ± 4.32 g). Cormel numbers was in the range of 2.67 ± 0.16 to 6.93 ± 0.71 with Megha Taro-2 exhibiting highest cormel numbers and Tamitim recorded the lowest. Corm yield was recorded to be in the range of 5.86 ± 1.79 t/ha to 21.02 ± 4.23 t ha^−1^ with the highest corm yield shown by genotype Tamachongkham and lowest in RC Taro-6. Cormel yield was recorded between the range 6.50 ± 3.57 and 20.19 ± 2.91 t ha^−1^ where genotype Megha Taro-1 displayed maximum cormel yield and Muktakeshi minimum. The total yield was observed maximum in genotype Tamitin (35.77 ± 0.90 t ha^−1^) and lowest (18.97 ± 7.45 t ha^−1^) in Rengama. This finding is similar to the findings of Khatemenla et al. (29) Kay (30) and Bekele and Boru (31). Thirugnanavel et al. 2015 (32) observed wide range of variations among the Colocasia germplasm for plant growth, no. of leaves, no. of suckers, leaf morphology, floral morphology, corm characters, yield characters, quality and Phytophthora leaf blight incidence. The differences in the corm weight and yield of taro may be attributed to the differences in accumulation of dry matter which been translocated to the corm, combined with a higher rate of yield-attributing characters, viz., plant height, leaf area etc., throughout growth, environmental conditions and the genetic makeup of the different genotype, which might have impacted the plant growth habit and number and size of corms and cormels (33, 34).

**Table 1:** Yield parameters of 30 genotypes of *Colocasia esculenta*.

Genotypes	Acc.	Corm weight (g)	Cormel weight (g)	Cormel nos./plants	Corm yield (t/ha)	Cormel yield (t/ha)	Total yield (t/ha)
BCC-2	1	208.33 ± 17.56 ^k-m^	63 ± 7.55^d-h^	5 ± 0.8^ab^	11.5 ± 3.77^ab^	13.66 ± 4.58^ab^	25.16 ± 7.03^a^
Kandha local	2	246.67 ± 12.58^h-l^	55 ± 9^e-m^	6.04 ± 0.94^ab^	14.8 ± 4.53^ab^	13.09 ± 5.26^ab^	27.89 ± 5.9^a^
Wahiajer local	3	344 ± 21.28^cd^	45.20 ± 6.75^f-n^	5.44 ± 0.76^ab^	16.22 ± 4.08^ab^	12.24 ± 3.79^ab^	28.46 ± 0.39^a^
Mairang local	4	310 ± 26.91^c-g^	85 ± 10^b-d^	4.12 ± 0.08^ab^	14.02 ± 4^ab^	19.37 ± 4.12^ab^	33.39 ± 6.99^a^
Thangitang	5	106.67 ± 15.28^o^	63.93 ± 8.67^d-g^	6 ± 1.73^ab^	10.06 ± 3.93^ab^	19.89 ± 4.98^ab^	29.95 ± 7.74^a^
RC Taro-6	6	100 ± 12^o^	56.67 ± 7.51^e-l^	5 ± 0^ab^	5.86 ± 1.79^b^	14.54 ± 4.06^ab^	20.4 ± 3.54^a^
AR3	7	128.33 ± 13.50^no^	44.67 ± 9.29^f-n^	5.2 ± 0.83^ab^	6.34 ± 2.49^b^	13.15 ± 4.31^ab^	19.49 ± 3.82^a^
ML2	8	316.67 ± 23.86^c-f^	51.73 ± 9.6^e-m^	4.78 ± 0.8^ab^	15.64 ± 3.11^ab^	14.5 ± 3.83^ab^	30.14 ± 6.12^a^
ML3	9	80 ± 16.09^o^	25.67 ± 8.74^no^	5 ± 0^ab^	9.32 ± 3.89^b^	10.3 ± 3.73^ab^	19.62 ± 0.29^a^
Naga local	10	283.33 ± 12.58^e-i^	61.67 ± 9.50^d-i^	4.07 ± 0.42^ab^	12.79 ± 2.82^ab^	12.78 ± 4.61^ab^	25.57 ± 4.02^a^
AR2	11	632 ± 14^a^	40.67 ± 6.03^g-o^	5.04 ± 1.88^ab^	14.33 ± 3.81^ab^	12.14 ± 4.78^ab^	26.47 ± 8.59^a^
C3	12	363.67 ± 12.58^c^	58 ± 6^e-k^	5 ± 0^ab^	17.24 ± 3.85^ab^	15.24 ± 4.66^ab^	32.48 ± 8.48^a^
C-14-9	13	170 ± 20^mn^	65.67 ± 6.03^d-f^	5 ± 0^ab^	8.84 ± 3.35^b^	16.07 ± 4.94^ab^	24.91 ± 1.63^a^
Tagitung white	14	287.33 ± 14.19^d-h^	38.67 ± 6.11^i-o^	5.05 ± 1.13^ab^	14.89 ± 3.31^ab^	13.82 ± 4.3^ab^	28.71 ± 6.92^a^
ML9	15	525 ± 15^b^	104 ± 9.54^b^	3.01 ± 0.06^b^	14.93 ± 3.74^ab^	17.37 ± 4^ab^	32.3 ± 10^a^
Tamakhan	16	104 ± 13.86^o^	37.33 ± 5.03^j-o^	5.76 ± 0.35^ab^	10.53 ± 3.92^ab^	14.81 ± 4.31^ab^	25.34 ± 0.42^a^
Naya bungalow	17	118.67 ± 22.03^no^	20.33 ± 4.51^o^	5.58 ± 2.73^ab^	9.5 ± 3.79^ab^	18.53 ± 3.62^ab^	28.02 ± 3.86^a^
Khweng-3	18	201.67 ± 17.56^lm^	35 ± 5^k-o^	5.29 ± 1.92^ab^	10.3 ± 2.55^ab^	14.47 ± 4.14^ab^	24.77 ± 5.22^a^
Tagitung purple	19	100 ± 15.10^o^	33.33 ± 5.03^l-o^	4.07 ± 0.16^ab^	13.98 ± 3.14^ab^	14.5 ± 3.76^ab^	28.48 ± 6.2^a^
Tamachongkham	20	641.67 ± 18.77^a^	71.67 ± 7.^64c-e^	3.72 ± 0.31^ab^	21.02 ± 4.23^a^	13.73 ± 3.29^ab^	34.75 ± 0.94^a^
Tamitin	21	625 ± 25^a^	151.67 ± 12.58^a^	2.67 ± 0.16^b^	16.47 ± 4.32^ab^	19.3 ± 3.9^ab^	35.77 ± 0.90^a^
Rengama	22	215 ± 20^j-m^	31 ± 5^m-o^	5 ± 0^ab^	9.75 ± 3.45^ab^	9.22 ± 4^ab^	18.97 ± 7.45^a^
Khweng-2	23	225 ± 23.90^i-m^	63 ± 8.19^d-h^	4.95 ± 2.37^ab^	9.93 ± 3.85^ab^	13.21 ± 4.15^ab^	23.14 ± 0.7^a^
White Gaurya	24	261 ± 23.26^f-k^	39 ± 6.56^h-o^	5 ± 0^ab^	15.13 ± 4.62^ab^	12.5 ± 3.79^ab^	27.62 ± 7.34^a^
Muktakeshi	25	251.67 ± 16.56^g-l^	19.53 ± 4.32^o^	5 ± 0^ab^	14.98 ± 4.56^ab^	6.5 ± 3.57^b^	21.48 ± 7.11^a^
IGB-5	26	241.67 ± 20.55^h-l^	45 ± 6^f-n^	4.89 ± 1.05^ab^	10.94 ± 1.27^ab^	11.62 ± 5.13^ab^	22.56 ± 4.22^a^
Megha Taro-1	27	270 ± 20^e-j^	59.67 ± 6.51^e-j^	5.08 ± 2.34^ab^	14.22 ± 1.95^ab^	20.19 ± 2.91^a^	34.41 ± 4.86^a^
Megha Taro-2	28	276 ± 17.09^e-i^	90.33 ± 10.02^bc^	6.93 ± 0.71^a^	14.47 ± 4.07^ab^	16.52 ± 4.17^ab^	30.99 ± 3.77^a^
Megha col	29	124 ± 10.15^no^	55.33 ± 6.11^e-l^	5.05 ± 0.93^ab^	8.47 ± 3.43^b^	14.38 ± 4.09^ab^	22.85 ± 7.5^a^
TBd 17–9	30	323.33 ± 22.48^c-e^	56.17 ± 5.34e^-l^	3.58 ± 0.08^ab^	10.66 ± 3.79^ab^	12.21 ± 4.15^ab^	22.87 ± 6.7^a^

Values given are mean (*n* = 3) with SD. Superscript lower case letters on each column designated statistical significance (*p* < 0.05).

### 3.2 Mineral composition

The mineral composition of the Colocasia genotypes is presented in Table 2. There were significant (*p* < 0.05) differences in mineral composition among the genotypes studied. The nitrogen (N) content ranged between 0.62 to1.79% with Tamitin genotype recording the highest N and least in Khweng-2. The phosphorus (P) content was registered highest in Tagitung white (0.49 ± 0.04%) and lowest (0.10 ± 0.02) in Megha Col. Further, the potassium (K) content ranges from (0.71 ± 0.02%) in Tagitung purple to (3.80 ± 0.03%) in Tamitin. The range in value of the minerals is probably due to the potential of each genotype to obtain nutrients from the soil (35). Buragohain et al. (36) reported similar findings.

**Table 2:** Mineral composition of the 30 genotypes of *Colocasia esculenta*.

Genotypes	N (%)	P (%)	K (%)	Ca + Mg%	Fe (ppm)	Zn (ppm)	Cu (ppm)	Mn (ppm)
BCC-2	1.58 ± 0.05^bc^	0.39 ± 0.04^a-d^	1.83 ± 0.03^d^	2.37 ± 0.04^a^	118 ± 5^a^	10.21 ± 1.95^kl^	3.24 ± 0.04^q^	22.56 ± 2.16^fg^
Kandha local	1.69 ± 0.05^ab^	0.36 ± 0.05^b-f^	1.54 ± 0.04^fg^	1.5 ± 0.03^g-i^	69.26 ± 4.95^mn^	23.68 ± 3.41^c-h^	4.23 ± 0.03^n^	44.62 ± 2.69^b^
Wahiajer local	1.41 ± 0.05^de^	0.35 ± 0.04^b-g^	1.26 ± 0.02^kl^	1.03 ± 0.03^q^	70.79 ± 3.84^l-n^	28.27 ± 2.11^b-f^	3.79 ± 0.04^o^	18.8 ± 3.33^f-i^
Mairang local	0.85 ± 0.04^i-k^	0.3 ± 0.04^c-h^	1.31 ± 0.01^jk^	1.66 ± 0.04^d^	89.55 ± 3.75^e-i^	28.92 ± 2.32^b-e^	2.67 ± 0.04^r^	19.74 ± 3.73^f-h^
Thangitang	1.55 ± 0.06^b-d^	0.35 ± 0.05^b-g^	1.11 ± 0.02^op^	1.3 ± 0.04^k-o^	90.74 ± 5.61^e-h^	31.72 ± 2.83^a-d^	5.78 ± 0.04^i^	10.58 ± 1.98^i^
RC Taro-6	1.25 ± 0.06^ef^	0.43 ± 0.05^ab^	3.62 ± 0.02^b^	1.6 ± 0.04^d-g^	78.64 ± 3.63^h-n^	20.54 ± 3.85^e-j^	3.81 ± 0.04^o^	15.34 ± 1.95^g-i^
AR3	0.64 ± 0.05^m^	0.28 ± 0.02^e-i^	1.37 ± 0.03^ij^	1.85 ± 0.03^c^	69.64 ± 4.39^mn^	18.33 ± 3.08^g-k^	6.16 ± 0.04^g^	12.36 ± 1.81^hi^
ML2	1.51 ± 0.06^cd^	0.29 ± 0.02^d-i^	1.58 ± 0.03^fg^	1.45 ± 0.03^h-j^	87.36 ± 4.57^e-j^	10.19 ± 1.96^kl^	7.51 ± 0.02^c^	18.62 ± 2.63^f-i^
ML3	1.24 ± 0.05^f^	0.25 ± 0.03^f-j^	1.13 ± 0.03^nop^	1.25 ± 0.05^m-o^	72.82 ± 3.5^j-n^	12.31 ± 1.9^j-l^	3.5 ± 0.04^p^	24.38 ± 2.9^e-g^
Naga local	1.22 ± 0.06^f^	0.43 ± 0.02^ab^	1.56 ± 0.03^fg^	1.12 ± 0.05^pq^	84.68 ± 4.48^f-l^	17.73 ± 3.65^g-l^	7.31 ± 0.04^d^	20.95 ± 3.44^f-h^
AR2	0.69 ± 0.06^lm^	0.43 ± 0.05^ab^	1.39 ± 0.02^i^	1.82 ± 0.03^c^	85.63 ± 4.5^f-k^	24.35 ± 1.95^b-g^	4.67 ± 0.03^k^	19.31 ± 3.3^f-i^
C3	1.26 ± 0.06^ef^	0.23 ± 0.03^h-j^	1.7 ± 0.03^e^	1.34 ± 0.05^j-n^	68.91 ± 4.6^mn^	19.23 ± 3.22^f-k^	2.54 ± 0.04^s^	20.57 ± 1.64^f-h^
C-14-9	0.63 ± 0.04^m^	0.19 ± 0.04^h-k^	1.59 ± 0.02^f^	1.2 ± 0.03^op^	76.04 ± 3.39^i-n^	12.87 ± 2.46^i-l^	3.15 ± 0.04^q^	24.68 ± 2.56^d-f^
Tagitung white	1.28 ± 0.05^ef^	0.49 ± 0.04^a^	1.9 ± 0.03^cd^	1.54 ± 0.03^f-h^	107.8 ± 4.33^a-c^	31.61 ± 3.55^a-d^	7.89 ± 0.04^b^	19.33 ± 2.11^f-i^
ML9	0.65 ± 0.05^m^	0.21 ± 0.04^h-j^	1.56 ± 0.02^fg^	1.61 ± 0.04^d-f^	84.75 ± 4.25^f-l^	27.94 ± 2.68^b-f^	5.16 ± 0.03^j^	33.24 ± 3.87^c-e^
Tamakhan	1.57 ± 0.06^bc^	0.40 ± 0.03^a-d^	1.23 ± 0.03^k-m^	1.8 ± 0.03^c^	76.04 ± 4.07^i-n^	30.5 ± 4.47^b-d^	4.38 ± 0.04^m^	33.47 ± 2.82^cd^
Naya bungalow	0.70 ± 0.05^klm^	0.38 ± 0.05^a-e^	1.51 ± 0.03^gh^	1.55 ± 0.03^e-h^	80.93 ± 4.46^g-m^	25.74 ± 3.66^b-g^	2.52 ± 0.04^s^	50.39 ± 2.85^ab^
Khweng-3	0.82 ± 0.05^i-l^	0.20 ± 0.05^h-k^	0.98 ± 0.02^r^	1.36 ± 0.04^j-l^	77.47 ± 4.31^h-n^	18.12 ± 3.05^g-k^	5.22 ± 0.05^j^	22.26 ± 2.84^fg^
Tagitung purple	0.9 ± 0.06^h-j^	0.15 ± 0.03^jk^	0.71 ± 0.02^s^	2.1 ± 0.03^b^	94.03 ± 4.75^c-g^	16.44 ± 1.69^g-l^	7.16 ± 0.04^e^	12.09 ± 2.01^hi^
Tamachongkham	1.19 ± 0.05^fg^	0.41 ± 0.03^a-c^	1.89 ± 0.03^cd^	1.35 ± 0.04^j-n^	112.78 ± 4.51^ab^	22.47 ± 3.46^d-h^	3.78 ± 0.04^o^	33.24 ± 3.94^c-e^
Tamitin	1.79 ± 0.05^a^	0.40 ± 0.02^a-d^	3.8 ± 0.03^a^	1.35 ± 0.02^j-m^	82.79 ± 4.65^f-m^	40.54 ± 4.70^a^	8.12 ± 0.04^a^	59 ± 4.07^a^
Rengama	0.75 ± 0.05^j-m^	0.25 ± 0.03^f-j^	1.07 ± 0.02^pq^	2.28 ± 0.03^a^	105.89 ± 4.49^a-d^	10.66 ± 3.46^kl^	4.22 ± 0.03^n^	25.61 ± 2.85^d-f^
Khweng-2	0.62 ± 0.06^m^	0.18 ± 0.03^i-k^	1.03 ± 0.02^qr^	1.26 ± 0.04^l-o^	72.38 ± 5.16^k-n^	17.68 ± 2.71^g-l^	6.48 ± 0.04^f^	22.09 ± 3.1^fg^
White Gaurya	0.97 ± 0.06^hi^	0.23 ± 0.03^h-j^	1.44 ± 0.03^hi^	1.4 ± 0.03^i-k^	84.53 ± 3.79^f-l^	22.27 ± 3.1^d-i^	5.93 ± 0.03^h^	22.49 ± 2^fg^
Muktakeshi	1.51 ± 0.06^cd^	0.30 ± 0.04^c-h^	1.95 ± 0.03^c^	1.07 ± 0.03^q^	95.19 ± 4.94^c-g^	14.25 ± 1.92^h-l^	2.3 ± 0.04^t^	12.38 ± 1.9^hi^
IGB-5	0.93 ± 0.06^hi^	0.24 ± 0.04^g-j^	1.31 ± 0.02^jk^	1.21 ± 0.04^op^	96.73 ± 4.22^c-f^	33.32 ± 3.1^ab^	4.55 ± 0.03^l^	22.38 ± 1.65^fg^
Megha Taro-1	1.4 ± 0.05^de^	0.45 ± 0.03^ab^	1.19 ± 0.03^l-n^	1.65 ± 0.04^de^	91.3 ± 5.06^d-h^	32.19 ± 2.03^a-c^	4.44 ± 0.04^lm^	35.56 ± 3.59^c^
Megha Taro-2	1.15 ± 0.06^fg^	0.25 ± 0.02^f-j^	1.17 ± 0.03^m-o^	1.4 ± 0.04^i-k^	101.06 ± 6.16^b-e^	29.71 ± 3.34^b-e^	6.17 ± 0.04^g^	26.24 ± 3.14^d-f^
Megha col	0.85 ± 0.05^i-k^	0.10 ± 0.02^k^	1.27 ± 0.02^kl^	1.24 ± 0.02^no^	65.4 ± 4.88^n^	8.33 ± 0.60^l^	2.09 ± 0.04^u^	12.17 ± 2.01^hi^
TBd 17–9	1.05 ± 0.05^gh^	0.48 ± 0.03^a^	1.71 ± 0.03^e^	1.8 ± 0.03^c^	105.72 ± 5.07^a-d^	33.44 ± 3.16^ab^	7.84 ± 0.04^b^	57.2 ± 2.97^a^

Values given are mean (*n* = 3) with SD. Superscript lower case letters on each column designated statistical significance (*p* < 0.05).

A diet rich in minerals such as potassium plays a crucial role in preventing hypertension, heart disease, stroke, renal damage, kidney stones, hypercalciuria, and osteoporosis (37). Additionally, potassium influences sugar metabolism, polymerization, and starch synthesis (38). In this study, calcium (Ca) and magnesium (Mg) content among the genotypes ranged from 1.03 ± 0.03% to 2.37 ± 0.04%, with BCC-2 recording the highest value and Wahiajer Local the lowest. Similarly, iron (Fe) content varied from 65.40 ± 4.88 ppm in Megha Col to 118 ± 5.00 ppm in BCC-2, aligning with findings reported by Khatemenla et al. (29). Iron is essential for photosynthesis and various metabolic processes in plants. In humans, iron deficiency is a leading cause of anemia, a widespread nutritional disorder globally. Additionally, inadequate iron intake has been linked to impaired brain function in infants, highlighting its critical role in human health (38).

Zinc (Zn) content was recorded highest in the genotype Tamitin (40.54 ± 4.70 ppm) and lowest in Megha Col. Similar findings were reported in a study by Brandao et al. (39). Zn is important for human growth and development because it synthesizes hormones and enzymes that promote mental and physical growth, helps in tissue repair, wound healing and other functions (40). Copper (Cu) is another essential mineral which is associated with the formation and growth of bones and absorption of iron during hemoglobin synthesis (41). From the present study it is found that the Cu content of the assessed genotypes ranges from (2.09 ± 0.04 ppm) in Megha Col to (8.12 ± 0.04 ppm) in Tamitin. Similarly, the study also revealed a wide range of variation in manganese (Mn) content of the genotypes evaluated where Tamitin recorded the highest value (59 ± 4.07 ppm). Lebot et al. (42) have also documented a high level of variability for the mineral contents as well as other nutrients in the South East Asian and Pacific taro germplasm. Furthermore, Boampong et al. (35) while highlighting the competitiveness of taro with other root and tuber crops reported significant genetic variations in mineral nutrients among the taro test genotypes in Ghana. The high content of essential elements such as K, Ca, Mg and Zn in the present accessions of taro can be an asset for alleviating hidden hunger especially in the rural and poor communities where access to a balanced diet is limited. Genetic differences may be attributed to the variations in mineral concentrations among the studied genotypes because the growing conditions (i.e., the same plot, the same planting distance, and the same planting date) were identical. Furthermore, Wills et al. (43) argued that variation in value for the minerals is probably due to the potential of each genotype to obtain nutrients from the soil.

### 3.3 Biochemical parameters

The maximum dry matter content was documented in Tamachongkham and least in Khweng-2 (Table 3). There were also a significant (*p* < 0.05) differences in starch content among different genotypes. Further, the highest starch content (35.49 ± 4.20%) was recorded in Khweng-2 while ML-9 recorded the lowest (15.16 ± 3%). This result is in line with the finding of 33. Varietal variation in starch and dry matter content in taro was also reported (43). Khweng-2 recorded the highest total sugar (5.60 ± 0.08%) and reducing sugar (3.74 ± 0.08%) while lowest total sugar (2.70 ± 0.07%) was recorded in Tamitin and reducing sugar in Naya Bungalow (1.68 ± 0.06%). Similar findings were reported by Sangeeta et al. 2023 (34) who stated that variation in sugar content could be due to the accumulation and translocation of photosynthates from leaves to fruits as carbohydrates are manufactured in the leaves. The degradation of insoluble polysaccharides and genetic makeup that result in the variable synthesis of total sugars, as well as variations in soil, environmental factors, and crop genetic makeup, could be the cause of the elevated level of total sugar. Total oxalate was found to be minimum (70.82 ± 5.99 mg/100 g dw) in C-3 and maximum (208.54 ± 5.21 mg/100 g dw) in Rengama. Soluble oxalate was also recorded in the range 18.52 ± 5.45 mg/100 g dw to 104 ± 3.40 mg/100 g dw where Rengama exhibited the maximum value. Insoluble oxalate ranged between 20 ± 4.84 mg/100 g dw and 160.32 ± 4.10 mg/100 g dw with genotype Muktakeshi showing the highest value. Similar findings were reported (44). Oxalate content is of interest because of its negative impact on nutrient bioavailability (45). The genotypes with lower oxalate content are desirable. The calcium oxalate content varies among different taro genotypes (33, 40). Since all genotypes were cultivated under similar climatic conditions, soil type, and cultivation practices, the observed variations in biochemical composition can be attributed primarily to genotypic differences. These inherent varietal differences ultimately influence the nutritional value of the crop (46).

**Table 3:** Biochemical parameters of the 30 genotypes of *Colocasia esculenta*.

Genotypes	Dry matter %	Starch %	Total Sugar %	Reducing Sugar %	Total Oxalate (mg/100 g) dw	Soluble Oxalate (mg/100 g) dw	Insoluble Oxalate (mg/100 g) dw	Crude Protein%	Crude Fiber %	Ash %
BCC-2	31.88 ± 4.36^ab^	19.23 ± 3.81^b-d^	3.05 ± 0.05^o^	1.75 ± 0.05^o^	85.78 ± 5.28^h-k^	34.42 ± 3.82^d-f^	51.36 ± 8.12^j-l^	3.25 ± 0.04^q^	3.06 ± 0.04^bcd^	4.65 ± 0.04^mn^
Kandha local	24.49 ± 4.48^ab^	27.19 ± 3.01^a-d^	4.95 ± 0.06^b^	3.29 ± 0.08^cd^	74.27 ± 4.05^kl^	28.6 ± 4.18^e-g^	45.68 ± 0.38^k-m^	5.7 ± 0.03^f^	2.91 ± 0.04^e-h^	7.4 ± 0.05^a^
Wahiajer local	28.44 ± 4.17^ab^	31.49 ± 3.74^a-c^	3.42 ± 0.07^lm^	3.25 ± 0.07^cd^	80.58 ± 4.5^i-l^	25.29 ± 5.13^f-g^	55.3 ± 4.92^i-l^	6.53 ± 0.05^d^	2.96 ± 0.04^d-g^	4.53 ± 0.04^n^
Mairang local	27.14 ± 4.95^ab^	28.93 ± 3.3^a-d^	3.42 ± 0.07^lm^	2.91 ± 0.06^fg^	94.42 ± 4.06^g-i^	35.72 ± 4.37^c-f^	58.69 ± 8.41^i-k^	5.65 ± 0.04^fg^	2.20 ± 0.02^p^	7.40 ± 0.05^a^
Thangitang	25.4 ± 4.16^ab^	20.39 ± 3.86^b-d^	3.97 ± 0.08^ef^	2.86 ± 0.06^f-h^	90 ± 5^h-j^	18.81 ± 4.46^g^	71.19 ± 8.08^hi^	5.77 ± 0.05^f^	2.96 ± 0.04^d-g^	6.49 ± 0.03^e^
RC Taro-6	35.25 ± 5.11^ab^	19.58 ± 4.36^b-d^	3.63 ± 0.07^h-k^	3.17 ± 0.08^de^	79.97 ± 4.35^i-l^	49.2 ± 4.4^c^	30.77 ± 4.16^mn^	5.69 ± 0.04^fg^	2.3 ± 0.04^op^	4.66 ± 0.05^mn^
AR3	28.12 ± 4.6^ab^	25.5 ± 4.92^a-d^	3.64 ± 0.09^h-k^	2.8 ± 0.08^f-i^	80.05 ± 4.81^i-l^	29.4 ± 5.18^d-g^	50.65 ± 0.39^j-l^	3.31 ± 0.04^q^	3.17 ± 0.04^ab^	6.41 ± 0.04^e^
ML2	29.35 ± 3.87^ab^	33.44 ± 4.9^ab^	3.52 ± 0.08^j-l^	2.66 ± 0.06^h-j^	92.58 ± 4.44^g-i^	34.5 ± 4.88^c-f^	58.08 ± 0.63^i-k^	5.76 ± 0.05^f^	2.97 ± 0.04^d-f^	7.22 ± 0.04^b^
ML3	26.18 ± 5.04^ab^	18.49 ± 4.75^cd^	3.71 ± 0.07^g-j^	3.01 ± 0.04^ef^	97.27 ± 3.81^gh^	28.32 ± 3.96^e-g^	68.96 ± 7.77^h-j^	5.57 ± 0.05^g^	3.19 ± 0.05^a^	4.8 ± 0.04^l^
Naga local	28.49 ± 5.02^ab^	25.32 ± 3.96^a-d^	3.46 ± 0.06^k-m^	3.25 ± 0.08^cd^	106.1 ± 4.9^g^	25.37 ± 4.8^fg^	80.73 ± 8.52^gh^	3.37 ± 0.05^pq^	2.92 ± 0.04^e-h^	6.98 ± 0.04^c^
AR2	32.41 ± 4.21^ab^	23.55 ± 5.21^a-d^	3.27 ± 0.08^mn^	2.03 ± 0.08^n^	136.76 ± 4.24^ef^	31.32 ± 5^d-g^	105.44 ± 4.43^d-f^	5.24 ± 0.05^h^	2.47 ± 0.05^mn^	4.7 ± 0.05^lm^
C3	25.11 ± 4.76^ab^	17.68 ± 5.67^cd^	4.79 ± 0.06^bc^	3.45 ± 0.07^bc^	70.82 ± 5.99^l^	23.89 ± 4.3^fg^	46.93 ± 1.74^k-m^	5.33 ± 0.04^h^	2.3 ± 0.04^op^	4.39 ± 0.04^o^
C-14-9	28.32 ± 5.02^ab^	24.91 ± 4.86^a-d^	3.5 ± 0.06^j-l^	1.8 ± 0.07^o^	76.43 ± 3.99^j-l^	36.18 ± 4.01^c-f^	40.25 ± 4.02^k-m^	3.44 ± 0.04^p^	2.87 ± 0.05^f-i^	5.04 ± 0.04^k^
Tagitung white	27.58 ± 4.53^ab^	27.7 ± 4.47^a-d^	3.74 ± 0.06^g-i^	2.15 ± 0.06^mn^	128.15 ± 4.56^f^	35.32 ± 4.76^c-f^	92.83 ± 4.98^fg^	6.31 ± 0.04^e^	2.81 ± 0.04^h-j^	4.18 ± 0.02^p^
ML9	28.54 ± 4.92^ab^	15.16 ± 3^d^	3.84 ± 0.06^e-h^	2.69 ± 0.06^g-j^	74.51 ± 3.9^kl^	32.26 ± 4.16^d-g^	42.25 ± 4.15^k-m^	5.28 ± 0.04^h^	3.14 ± 0.04^a-c^	5.2 ± 0.04^j^
Tamakhan	35.29 ± 5.04^ab^	25.52 ± 4.49^a-d^	3.46 ± 0.04^k-m^	2.63 ± 0.07^ij^	85.28 ± 4.61^h-l^	65.27 ± 5.77^b^	20 ± 4.84^n^	3.44 ± 0.04^p^	2.84 ± 0.03^g-i^	4.19 ± 0.04^p^
Naya bungalow	34.05 ± 7.01^ab^	31.27 ± 5.16^a-c^	3.15 ± 0.07^no^	1.68 ± 0.06^o^	196.87 ± 4.5^ab^	36.97 ± 4.3^c-f^	159.9 ± 7.78^a^	6.48 ± 0.05^d^	2.37 ± 0.04^no^	5.43 ± 0.04^i^
Khweng-3	34.64 ± 6.05^ab^	26.04 ± 4.55^a-d^	4 ± 0.07^de^	3.17 ± 0.08^de^	73.93 ± 4.61^kl^	34.68 ± 4.55^c-f^	39.25 ± 4.94^lm^	4.66 ± 0.03^ij^	2.76 ± 0.04^ij^	4.06 ± 0.04^p^
Tagitung purple	30.77 ± 4.76^ab^	20.73 ± 4.52^b-d^	3.78 ± 0.06^f-h^	2.63 ± 0.06^ij^	134.47 ± 4.17^ef^	29.26 ± 4.78^d-g^	105.2 ± 4.32^d-f^	3.6 ± 0.03^o^	2.23 ± 0.03^p^	5.64 ± 0.04^gh^
Tamachongkham	36.93 ± 3.83^a^	20.43 ± 3.98^b-d^	3.51 ± 0.07^j-l^	2.89 ± 0.06^fg^	193.14 ± 3.88^b^	41.76 ± 4.37^c-e^	151.38 ± 0.65^ab^	6.49 ± 0.04^d^	2.7 ± 0.04^jk^	6.18 ± 0.05^f^
Tamitin	23.29 ± 5.21^ab^	19.24 ± 5.53^b-d^	2.7 ± 0.07^p^	1.79 ± 0.07^o^	187.29 ± 4.54^bc^	102.46 ± 4.34^a^	84.82 ± 7.3^gh^	4.76 ± 0.05^i^	2.62 ± 0.04^kl^	6.68 ± 0.04^d^
Rengama	21.12 ± 4.6^b^	20.56 ± 4.74^b-d^	4.63 ± 0.06^c^	3.34 ± 0.07^cd^	208.54 ± 5.21^a^	104 ± 3.40^a^	104.54 ± 7.79^d-f^	7.10 ± 0.04^a^	3.23 ± 0.04^a^	7.26 ± 0.04^b^
Khweng-2	20 ± 3.39^b^	35.49 ± 4.20^a^	5.6 ± 0.08^a^	3.74 ± 0.08^a^	165.96 ± 5.34^d^	30.79 ± 4.45^d-g^	135.16 ± 8.82^bc^	3.79 ± 0.04^n^	3.04 ± 0.04^c-e^	3.90 ± 0.08^q^
White Gaurya	26.74 ± 4.37^ab^	30.46 ± 4.17^a-c^	3.85 ± 0.07^e-g^	3.23 ± 0.08^d^	174.18 ± 3.78^cd^	24.25 ± 4.72^fg^	149.93 ± 7.39^ab^	4.58 ± 0.05^jk^	2.41 ± 0.03^no^	5.61 ± 0.04^h^
Muktakeshi	23.26 ± 4.62^ab^	24.44 ± 5.11^a-d^	3.16 ± 0.09^no^	2.37 ± 0.07^kl^	186.52 ± 3.99^bc^	26.21 ± 4.81^fg^	160.32 ± 4.10^a^	4.26 ± 0.04^m^	3.12 ± 0.04^a-c^	6.19 ± 0.05^f^
IGB-5	28.54 ± 4.85^ab^	17.35 ± 5.06^cd^	3.76 ± 0.07^g-i^	2.65 ± 0.08^h-j^	141.16 ± 4.54^ef^	18.52 ± 5.45^g^	122.64 ± 0.94^cd^	6.74 ± 0.04^c^	2.62 ± 0.04^kl^	7.35 ± 0.04^ab^
Megha Taro-1	31.37 ± 4.79^ab^	31.28 ± 3.85^a-c^	2.74 ± 0.06^p^	2.25 ± 0.05^lm^	142.75 ± 4.72^e^	32.95 ± 4.25^d-g^	109.8 ± 7.74^def^	4.52 ± 0.05^kl^	2.57 ± 0.08^lm^	5.65 ± 0.04^gh^
Megha Taro-2	27.69 ± 4.12^ab^	30.41 ± 4.95^a-c^	3.2 ± 0.06^no^	1.81 ± 0.06^o^	148.54 ± 4.58^e^	35.22 ± 5.05^c-f^	113.32 ± 4.69^de^	6.94 ± 0.05^b^	2.58 ± 0.04^k-m^	6.39 ± 0.05^e^
Megha col	31.08 ± 5.57^ab^	18.3 ± 5.41^cd^	4.2 ± 0.06^d^	3.64 ± 0.07^ab^	165.37 ± 4^d^	43.82 ± 4.28^cd^	121.55 ± 7.23^cd^	7.09 ± 0.04^a^	2.95 ± 0.04^d-g^	6.88 ± 0.05^c^
TBd 17–9	27.48 ± 4.06^ab^	21.41 ± 4.97^a-d^	3.56 ± 0.06^i-l^	2.58 ± 0.08^jk^	134.62 ± 4.56^ef^	35.36 ± 4.69^c-f^	99.27 ± 4.8^e-g^	4.43 ± 0.04^l^	2.6 ± 0.04^kl^	5.77 ± 0.05^g^

N, Nitrogen; P, Phosphorus; K, Potassium; Ca, calcium; Mg, Magnesium; Fe, Iron; Zn, Zinc; Cu, Copper; Mn, Manganese. Values given are mean (*n* = 3) with SD. One way analysis of variance (ANOVA) plus post-hoc Tukey test was done to compare means. Superscript lower case letters on each column designated statistical significance (*p* < 0.05).

### 3.4 Crude protein, crude fiber and ash content

Data in Table 3 indicated significant variation in crude protein, crude fiber, and ash content among taro genotypes. Crude protein content ranged 3.25 ± 0.04% to 7.10 ± 0.04% with BCC-2 recorded the lowest and Rengama the highest, showcasing variability across different genotypes. Notably, the observation in our study align closely with those reported for other taro variety (47). Fagbemi and Olaofe (48) reported that taro can be an excellent source of protein to children who are sensitive to milk. Crude fiber ranged between 2.20 ± 0.02% to 3.23 ± 0.04% with maximum value shown by Rengama and minimum in Mairang local. This findings corresponds to the report (35, 49). The findings are important as crude fiber provides roughages that aid digestion (50). Ash content varied considerably from 3.90 ± 0.08% in Khweng-2 to 7.40 ± 0.05% in Mairang Local. These results are in agreement with the work done (49, 51, 52). This ash content may indicate that these samples could contain substantial amounts of dietary minerals, as confirmed in an earlier report (47).

### 3.5 Antioxidant activity

#### 3.5.1 FRAP assay

FRAP antioxidants capacity is a simple and inexpensive assay that offers a putative index of the potential antioxidant activity of plant materials. Principally, the FRAP assay treats the antioxidants in the sample as reductants in a redox-linked colourimetric reaction. The reducing power assay, i.e., the transformation of Fe3C to Fe2C in the presence of either the extract or the standard (ascorbic acid), is a measure of reducing capability (53). Our study as presented in Table 4 indicated that among all the genotypes studied, the FRAP values recorded highest in Rengama (52 ± 4.55 mM FeSO_4_E/100 g dw), while the lowest value was recorded in Tamakhan (35 ± 4.13 mM FeSO_4_E/100 g dw). Similar FRAP value was recorded in a study (54). The variation may be related to genotypic differences, environmental factors, and methods used for determination (55).

**Table 4:** Antioxidant activity of 30 genotypes of *Colocasia esculenta*.

Genotypes	FRAP (mM FeSO_4_E/100 g) dw	DPPH (IC_50_) sample (mg/ml) dw	Total phenolic content (mgGAE/100 g) dw	Total anthocyanin content (mg/100 g) dw
BCC-2	38.09 ± 3.28^b-d^	1.13 ± 0.07^a^	91.78 ± 3.4^a-e^	0.50 ± 0.04^k^
Kandha local	36.91 ± 3.28^cd^	1.12 ± 0.05^a^	86.19 ± 3.95^b-f^	0.73 ± 0.04^a-i^
Wahiajer local	48.13 ± 3.99^a-d^	1.01 ± 0.05^a-f^	84.02 ± 3.55^c-f^	0.66 ± 0.05^e-j^
Mairang local	40.74 ± 4.1^a-d^	0.83 ± 0.05^g-i^	94.17 ± 4^a-d^	0.55 ± 0.05^jk^
Thangitang	47.75 ± 4.64^a-d^	1 ± 0.05^a-f^	81.93 ± 3.35^d-f^	0.74 ± 0.04^a-i^
RC Taro-6	48.01 ± 4.45^a-d^	1.09 ± 0.05^ab^	84.37 ± 4^c-f^	0.62 ± 0.05^h-k^
AR3	45.2 ± 4.63^a-d^	0.82 ± 0.04^g-i^	91.64 ± 3.44^a-e^	0.72 ± 0.05^a-i^
ML2	50.92 ± 3.59^ab^	0.72 ± 0.05^i^	97.01 ± 4.18^a-c^	0.67 ± 0.04^d-j^
ML3	36.27 ± 4.18^cd^	1.07 ± 0.06^ab^	88.79 ± 3.46^a-f^	0.81 ± 0.05^a-d^
Naga local	47.87 ± 4.51^a-d^	0.83 ± 0.05^g-i^	95.1 ± 4.62^a-d^	0.73 ± 0.05^a-i^
AR2	49.96 ± 4.22^a-c^	0.89 ± 0.05^d-h^	84.55 ± 4.45^c-f^	0.84 ± 0.05^ab^
C3	40.85 ± 4.22^a-d^	0.95 ± 0.05^b-g^	94.91 ± 4.4^a-d^	0.75 ± 0.06^a-h^
C-14-9	36.9 ± 4.14^cd^	1.09 ± 0.06^ab^	80.22 ± 3.32^ef^	0.64 ± 0.05^f-j^
Tagitung white	45.76 ± 4.52^a-d^	0.72 ± 0.06^i^	95.09 ± 3.3^a-d^	0.76 ± 0.05^a-g^
ML9	37.72 ± 4.93^b-d^	1.05 ± 0.06^a-c^	83.71 ± 4.26^d-f^	0.68 ± 0.04^c-j^
Tamakhan	35 ± 4.13^d^	1.13 ± 0.05^a^	82.67 ± 4.47^d-f^	0.64 ± 0.04^g-k^
Naya bungalow	43.24 ± 3.41^a-d^	0.86 ± 0.06^e-i^	90.63 ± 3.69^a-f^	0.7 ± 0.05^b-i^
Khweng-3	49.64 ± 4.39^a-c^	0.73 ± 0.04^i^	94.52 ± 4.45^a-d^	0.82 ± 0.04^a-c^
Tagitung purple	44.1 ± 4.06^a-d^	0.95 ± 0.05^b-g^	94.86 ± 4.55^a-d^	0.71 ± 0.05^a-i^
Tamachongkham	49.61 ± 4.53^a-c^	1.02 ± 0.05^a-e^	98.98 ± 4.33^ab^	0.78 ± 0.05^a-f^
Tamitin	40.68 ± 4.02^a-d^	0.86 ± 0.05^f-i^	92.92 ± 3.97^a-e^	0.8 ± 0.05^a-e^
Rengama	52 ± 4.55^a^	0.71 ± 0.06^i^	100 ± 4.88^a^	0.86 ± 0.05^a^
Khweng-2	46.14 ± 5.18a^-d^	0.72 ± 0.06^i^	94.3 ± 5.01^a-d^	0.84 ± 0.05^ab^
White Gaurya	40.7 ± 3.89^a-d^	1.11 ± 0.05^a^	86.14 ± 4.74^b-f^	0.82 ± 0.04^a-c^
Muktakeshi	46.11 ± 4.85^a-d^	1.03 ± 0.04^a-d^	78 ± 4.08^f^	0.74 ± 0.05^a-i^
IGB-5	45.04 ± 4.54^a-d^	0.76 ± 0.05^hi^	90.57 ± 4.24^a-f^	0.79 ± 0.06^a-f^
Megha Taro-1	36.43 ± 4.61^cd^	1.08 ± 0.05^ab^	83.68 ± 3.14^d-f^	0.6 ± 0.05^i-k^
Megha Taro-2	42.81 ± 4.47^a-d^	0.94 ± 0.05^b-g^	80.21 ± 4.58^ef^	0.66 ± 0.05^e-j^
Megha col	42.66 ± 3.91^a-d^	1.07 ± 0.05^a-c^	93.09 ± 4.34^a-e^	0.77 ± 0.05^a-g^
TBd 17–9	41.38 ± 4.23^a-d^	0.91 ± 0.04^c-h^	93.69 ± 4.49^a-d^	0.63 ± 0.05^g-k^

Values given are mean (*n* = 3) with SD. Superscript lower case letters on each column designated statistical significance (*p* < 0.05).

#### 3.5.2 DPPH radical scavenging activity assay

The free radical chain reaction is widely accepted as the most important mechanism of lipid peroxidation. Radical scavengers terminate the peroxidation chain reaction by directly counteracting and quenching peroxide radicals. The capacity of polyphenols to transport labile H atoms to radicals is a probable mechanism of antioxidant protection, which can be assessed universally and rapidly using DPPH. Furthermore, DPPH is the most common and cost-effective way to determine the free radical scavenging capacity of natural products, which are major factors in biological damage caused by oxidative stress (56). In the present study we was observed that IC_50_ values ranged from 0.71 ± 0.06 mg/mL dw in Rengama to 1.13 ± 0.07 mg/mL dw in BCC-2 which indicatesthat all the taro genotypes exhibited antioxidant activity, although there were differences in the extent of this property between the genotypes (*p* < 0.05). This observation was in line with the results reported in previous studies by Gonçalves et al. (57). Ali et al. (58) reported that lower IC_50_ value reflects better DPPH radical scavenging activity. Therefore,Rengama with least IC_50_ may be considered to have higher antioxidant activity or better radical scavenging activity than BCC-2 with highest IC_50_ value as argued by Mariod et al. (59).

#### 3.5.3 Total phenolic content

Phenolic compounds may act as antioxidants, attractants, structural polymers, and signal compounds. They prevent oxidative damage to biomolecules due to the chelating of metal ions that cause the production of free radicals (60). Our results indicated the presence of significant variation in total phenol content among the *Colocasia* genotypes. Total phenolic content varied significantly from 78 ± 4.08 mgGAE/100 g dw in Muktakeshi to 100 ± 4.88 mgGAE/100 g dw in Rengama. Notably, Ouédraogo et al. (61) reported similar observation with respect to total phenolic content of taro. This variation in phenolic content could be attributed to environmental factors, extraction conditions, genetic factors, existence of distinct phenolic compounds and analytical method (61–64).

#### 3.5.4 Anthocyanin content

Anthocyanins are water-soluble and vacuolar pigments found in most species in the plant kingdom. It plays a role in preventing, ameliorating, and scrubbing oxidative stress, thus retarding several diseases and physiological malfunctions (65). A significant variation in anthocyanin content was observed among the genotypes. The anthocyanin content ranged 0.50 ± 0.04 mg/100 g dw to 0.86 ± 0.05 mg/100 g dw with Rengama recorded maximum and BCC-2 the minimum. Our finding is in line with Das et al. (66) who reported similar anthocyanin content in raw taro powder. This difference in anthocyanin content might be due to the effects of genetics, agro-ecological conditions such as pH, light, temperature, and horticultural practices (67).

### 3.6 Correlation among yield and quality traits

Correlation analysis is a crucial statistical approach used to understand the interrelationships among multiple traits in plant breeding and agronomic research. In this study, Pearson’s correlation coefficient was employed to quantify the strength and direction of associations between yield related traits, biochemical parameters, and antioxidant properties in Colocasia. The rationale for implementing correlation analysis lies in its ability to identify key traits that influence overall crop performance, allowing breeders and researchers to make informed decisions in trait selection and genetic improvement programs. By analyzing correlation patterns, we can determine whether improvements in a particular trait, such as yield, have a direct or indirect effect on other parameters, including nutritional and antioxidant properties. A strong positive correlation suggests that traits can be co-selected for genetic enhancement, whereas a negative correlation indicates potential trade-offs that must be considered in breeding strategies. Additionally, correlation analysis provides insights into the physiological and biochemical linkages among traits, revealing how metabolic pathways influence yield and quality attributes. For instance, the accumulation of starch and sugars in corms may affect other biochemical properties such as phenolic content and antioxidant activity, which are crucial for both nutritional value and storage quality.

**Figure 1:**
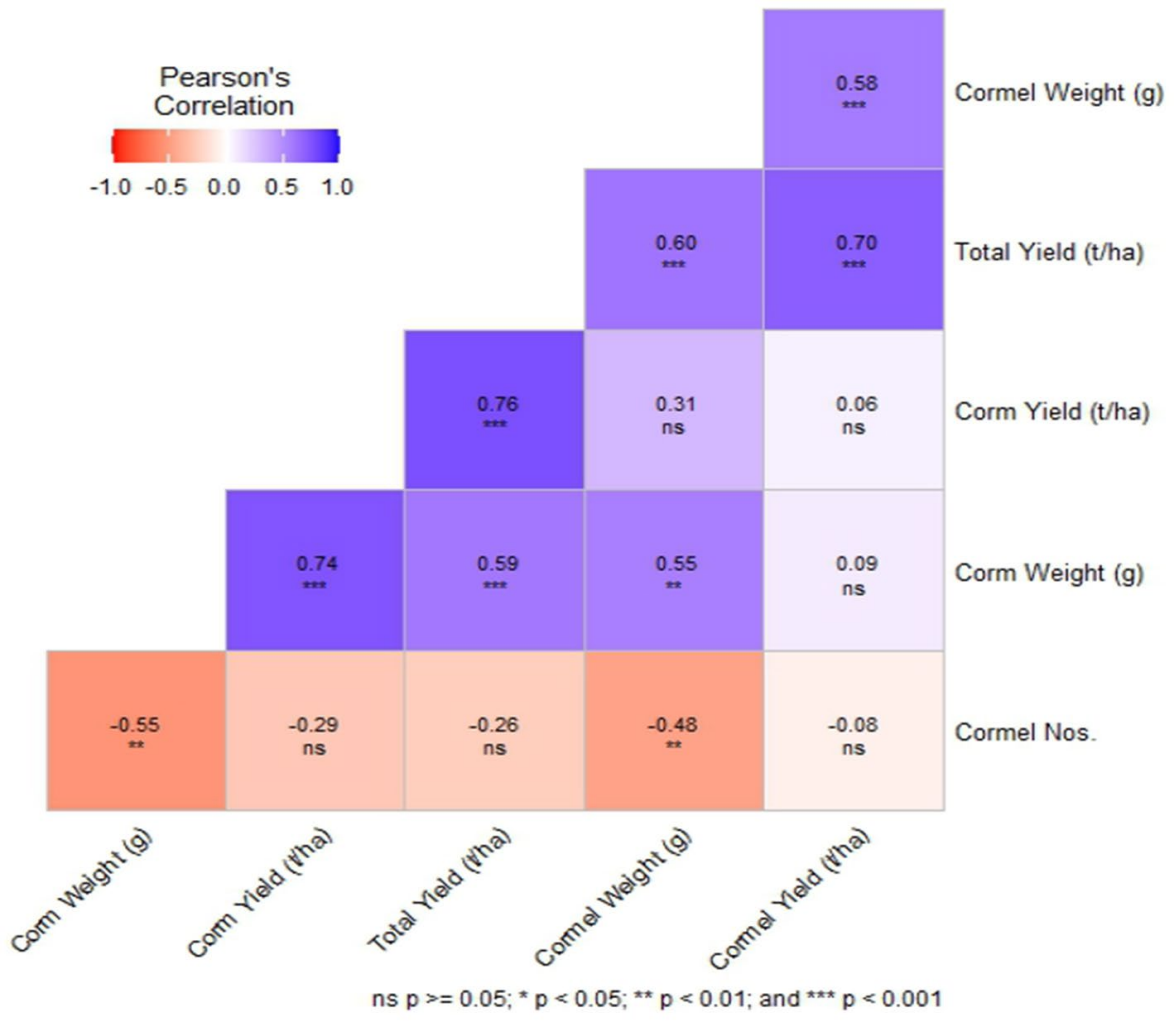
A Pearson’s correlation heatmap illustrating the correlation among the yield parameters of *Colocasia esculenta* L. genotypes. Different colors represent negative (red) to positive (blue) correlation.

The total yield exhibited a significant positive correlation with corm yield, cormel yield, cormel weight, and corm weight, suggesting that these yield-related traits are interdependent and can be used as selection criteria for improving overall productivity (Figure 1). This is in accordance with the study (68). Likewise, the correlation coefficient of all the other yield related traits is presented in the heatmap below (Figure 1) further illustrates the strength and direction of these relationships.

**Figure 2:**
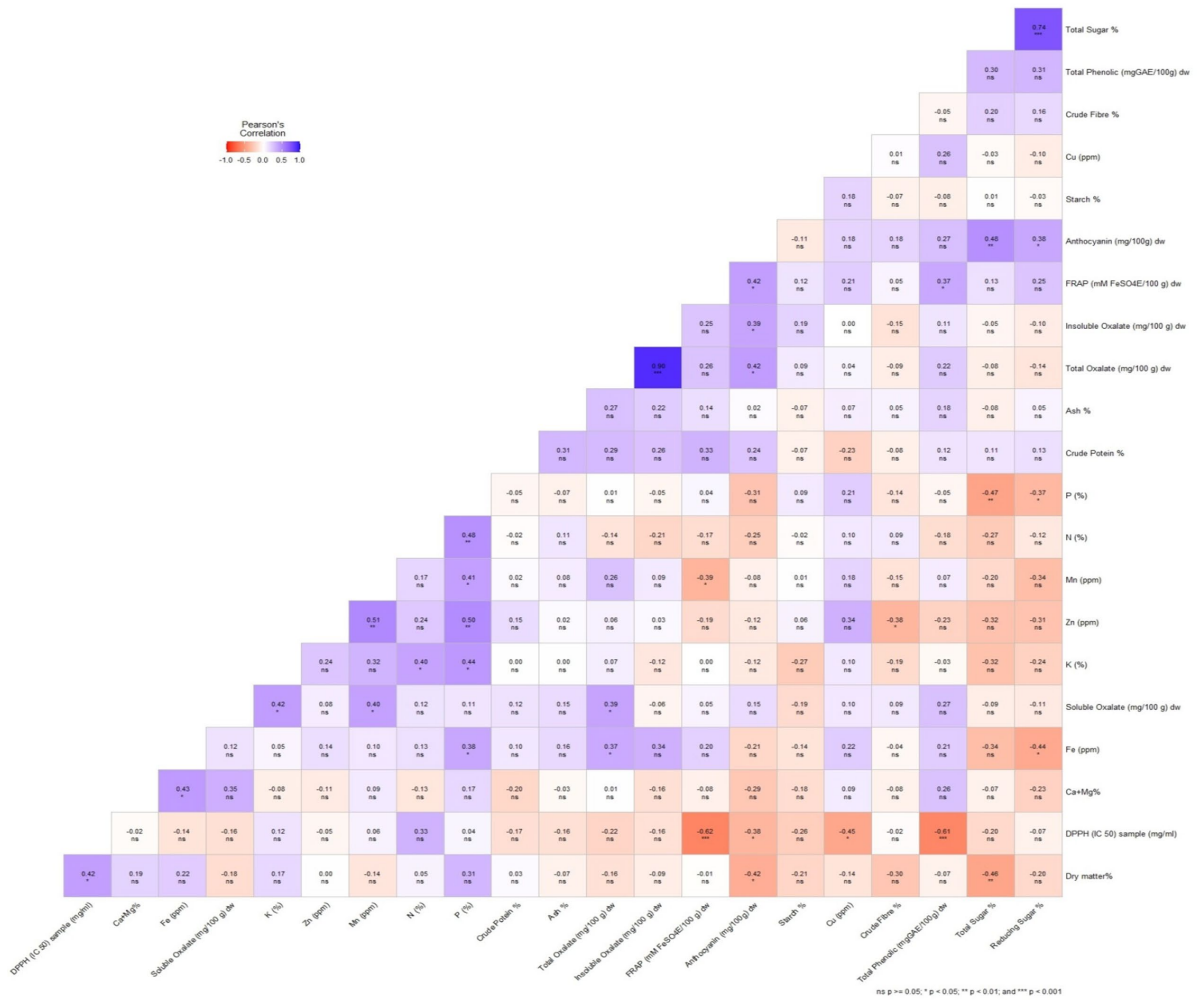
A Pearson’s correlation heatmap illustrating the correlation among the biochemical and antioxidant parameters of *Colocasia esculenta* L. genotypes. Different colors represent negative (red) to positive (blue) correlation.

Among quality traits, dry matter content showed a significant positive correlation with DPPH but a significant negative correlation with anthocyanin and total sugar (Figure 2). This indicates that higher dry matter content may enhance antioxidant activity (DPPH), while possibly reducing anthocyanin and sugar accumulation. However, starch content which is the most desirable quality trait in Colocasia showed positive but non-significant correlation with insoluble oxalate, Cu, FRAP, total oxalate, P, Zn, Mn and total sugar and negative non-significant correlation with the other quality parameters (Figure 2). This suggests that while starch accumulation is largely independent of these traits, external factors such as genetic variation and environmental conditions may play a crucial role in starch biosynthesis. A study (61) reported no significant correlation between starch and antioxidant properties (DPPH, FRAP) or sugar content. Additionally, there was no significant positive correlation between crude protein and mineral content (Figure 2), suggesting that selecting for high protein does not necessarily result in increased mineral levels. This finding aligns with a study by Boampong et al. (35), which also reported that most minerals did not show a significant correlation with protein content.

With respect to antioxidants parameters, total phenolic content exhibited the highest positive significant correlation with FRAP (*r* = 0.37, *p* < 0.05) and highly significant negative correlation with DPPH (*r* = −0.61, *p* < 0.001). FRAP had significant positive correlation with anthocyanin (*r* = 0.42, *p* < 0.05) and a highly significant negative correlation with DPPH (*r* = −0.62, *p* < 0.001). A significant negative correlation was observed between DPPH and anthocyanin (*r* = −0.38, *p* < 0.05; Figure 2). Makori et al. (69) also reported a similar positive correlation between total phenolic content and FRAP (r^2^ = 0.535, *p* < 0.001). Previous studies have also reported a positive correlation between antioxidant activity and both phenolic and anthocyanin content (70, 71), highlighting the crucial role of polyphenols in plant extracts in determining their antioxidant potential (72). This correlation serves as a reliable indicator of the antioxidant properties of plant-based compounds (73). By understanding these correlations, breeding programs can prioritize traits that contribute to both yield and quality, ensuring the development of high-yielding Colocasia cultivars with enhanced nutritional and functional properties.

### 3.7 Principal component analysis

The principal component analysis (PCA), a multivariate statistical technique, was used in the current study for: (i) dimension reduction or selection of a minimum data set (MDS) and (ii) computation of weights (Wi). The system attributes are best represented by the principal components with high eigenvalues and variables with high factor loadings. Therefore, we examined only the PCs with eigenvalues ≥1.0. PCA helps in understanding the underlying structure of a complex dataset by transforming correlated variables into a set of uncorrelated components ranked by their contribution to overall variance. This transformation allows for better interpretation of the relationships among yield-related and biochemical parameters, enabling the identification of key traits that significantly influence Colocasia productivity and quality.

**Figure 3:**
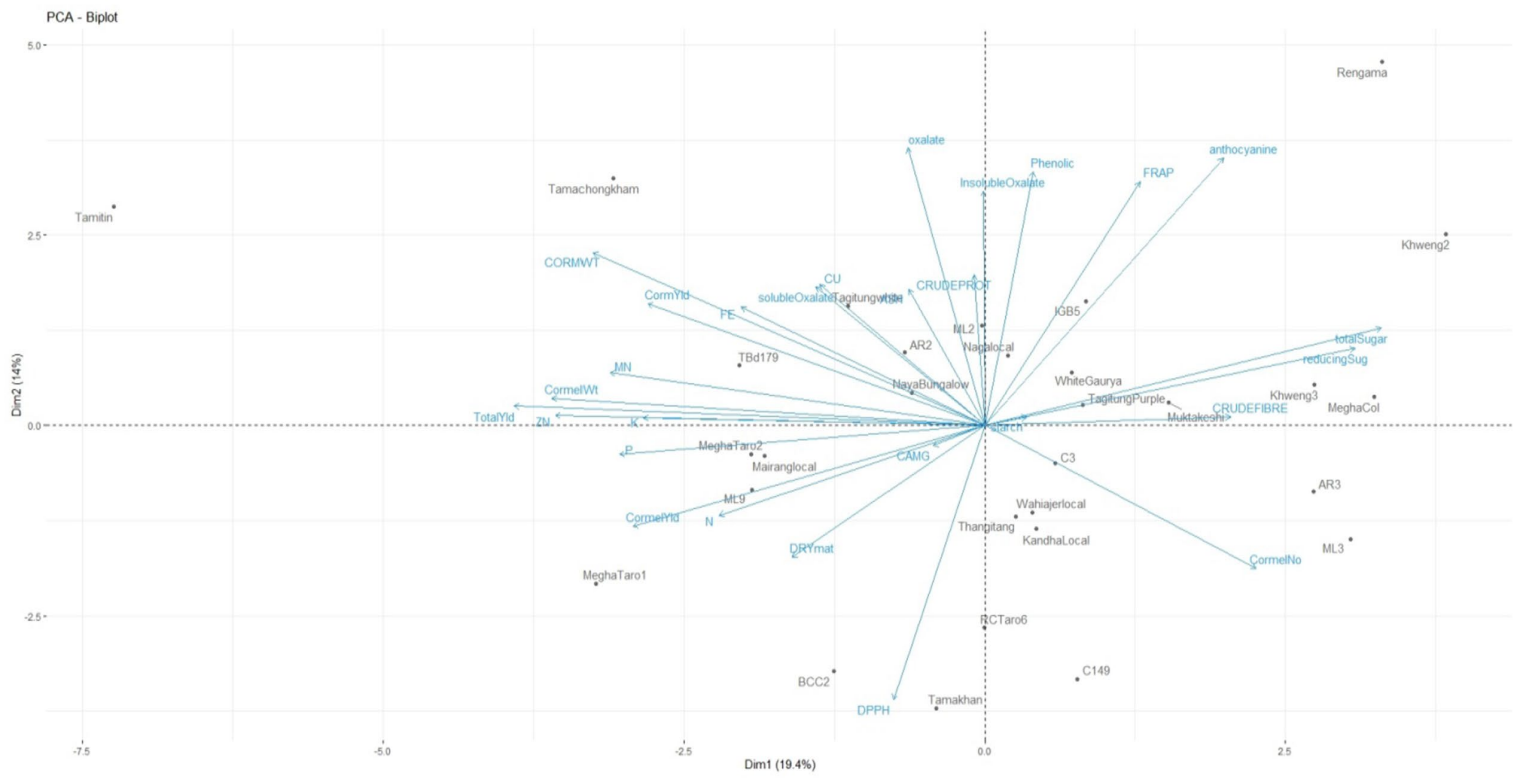
Genotype-by-trait biplot analysis for two principal components of *Colocasia esculenta* L. genotypes.

Phenotypic data of 30 *Colocasia esculenta* L. genotypes for yield and related traits were utilized for generating genotype-by-trait biplot graphs (Figure 3) and analyzed using the first two principal components. PC1 explained 19.4% of the total variation, while PC2 accounted for 14%. Among the 28 yield-contributing and biochemical traits, total sugar, reducing sugar, cormel numbers, crude fiber, anthocyanin, and FRAP contributed maximally to the diversity in PC1 (Table 5). PC1 primarily explains the trade-off between yield and biochemical properties such as sugar content and fiber accumulation. The selection of genotypes with high yield traits should consider their sugar accumulation, as higher sugar content may indicate enhanced palatability but could inversely impact dry matter content and storage quality. The significant contribution of FRAP suggests that yield improvements must be balanced with maintaining antioxidant potential for enhanced nutritional quality. Similarly, total oxalate, anthocyanin, total phenolics, FRAP, insoluble oxalate, corm weight, crude protein, soluble oxalate, Cu, ash, corm yield, Fe, total sugar, and reducing sugar exhibited maximum diversity in PC2. PC2 represents biochemical and anti-nutritional components, indicating that genotypes with higher antioxidant activity tend to accumulate phenolic compounds and oxalates. While high phenolic content is beneficial for human health, excessive oxalate levels can hinder mineral bioavailability. Thus, breeders should focus on achieving an optimal balance between antioxidant properties and oxalate content for developing nutritionally superior Colocasia varieties.

**Table 5:** Principal component analysis of *Colocasia esculenta* L. genotypes.

PCs	PC1	PC2	PC3	PC4	PC5	PC6
% variance expected	19.4	14	8.6	8.3	6.8	6.3
Corm weight (g)	−0.26	0.22	−0.09	0.07	−0.18	0.18
Cormel weight (g)	−0.29	0.03	−0.13	**0.33**	−0.03	−0.001
Cormel nos.	0.19	−0.18	−0.16	−0.30	0.12	−0.18
Corm yield (t/ha)	−0.23	0.15	−0.24	−0.09	−0.18	0.24
Cormel yield (t/ha)	−0.24	−0.13	−0.23	0.06	0.25	0.03
Total yield (t/ha)	−0.32	0.02	−0.33	−0.03	0.04	0.19
Dry matter %	−0.13	−0.17	0.18	−0.19	−0.20	**0.41**
Starch %	0.03	0.01	−0.25	−0.25	**0.42**	−0.10
Total sugar %	**0.27**	0.12	−0.18	0.25	0.02	0.02
Reducing sugar %	0.25	0.10	−0.18	0.24	−0.14	0.16
Total oxalate (mg/100 g) dw	−0.05	**0.35**	0.07	−0.26	−0.13	−0.30
Soluble oxalate (mg/100 g) dw	−0.12	0.18	0.25	0.24	−0.03	−0.31
Insoluble oxalate (mg/100 g) dw	−0.001	0.30	−0.05	−0.40	−0.12	−0.17
FRAP (mM FeSO_4_E/100 g) dw	0.11	0.31	0.05	−0.13	0.05	0.25
DPPH (IC 50) sample (mg/ml dw)	−0.06	−0.35	−0.01	−0.05	−0.40	−0.09
Total phenolic (mgGAE/100 g) dw	0.03	0.32	0.16	0.17	0.13	0.27
Anthocyanin (mg/100 g) dw	0.16	0.34	−0.13	0.09	−0.12	−0.13
N %	−0.18	−0.11	−0.02	0.02	−0.15	−0.12
P %	−0.25	−0.04	0.20	−0.13	0.09	−0.04
K %	−0.23	0.01	0.15	0.20	−0.21	−0.12
Ca + Mg %	−0.04	−0.03	**0.45**	0.002	0.21	0.11
Fe (ppm)	−0.17	0.15	0.32	−0.27	0.03	0.13
Zn (ppm)	−0.29	0.01	−0.18	−0.02	0.17	−0.15
Cu (ppm)	−0.11	0.18	0.03	0.15	0.41	0.05
Mn (ppm)	−0.26	0.07	0.03	0.12	0.06	−0.37
Crude protein %	−0.01	0.19	−0.18	−0.17	−0.23	−0.02
Crude fiber %	0.17	0.01	0.12	0.15	−0.10	−0.19
Ash %	−0.05	0.17	−0.02	−0.02	−0.11	−0.06

Bold values indicates traits contributing maximum towards respective PCs.

The positioning of genotypes in different quadrants of the biplot graph suggests distinct trait associations. For instance, genotypes such as Tamitin, Tamachongkham, Tagitung, and AR2 in the first (top left) quadrant exhibited high loadings for yield-contributing and mineral traits like corm weight, corm yield, Mn, Zn, and K, suggesting potential for high productivity. Meanwhile, Rengama, Khweng-2, and IGB-5 in the second (top right) quadrant contains seven variables such as anthocyanin, FRAP, phenolic, total sugar, reducing sugar, crude fiber and starch. The genotypes positioned in these quadrants suggest potential breeding lines for different end uses. High-yielding genotypes are suitable for commercial production, while those rich in antioxidants are valuable for nutraceutical applications. This classification aids in targeted breeding strategies to meet diverse consumer and industrial demands. Genotypes in the third (bottom right) quadrant, such as C3, AR3, Wahiajer Local, Thangitang, Kandha Local, and ML3, primarily influenced cormel numbers, which could impact propagation efficiency. Higher cormel numbers indicate superior propagation ability, which is beneficial for mass multiplication and commercial production. However, trade-offs with yield and biochemical traits should be carefully evaluated to ensure that productivity is not compromised. The fourth (bottom left) quadrant, containing Megha Taro-2, Mairang Local, and ML9, was associated with P, cormel yield, N, Ca + Mg, dry matter, and DPPH, highlighting genotypes with higher nutrient density and stress resilience. These genotypes exhibit superior nutrient profiles and stress tolerance, making them ideal candidates for improving soil nutrient efficiency and resilience under variable environmental conditions. Selection of such genotypes can contribute to sustainable agricultural practices and improved food security. The identified traits within the axes of the first five PCs with maximum variance exhibited significant influence on the phenotypes of the Colocasia genotypes evaluated and could efficiently be utilized for future selection among these genotypes. The study revealed distribution of variance over multiple PCs, which may be attributed to the poor correlation among yield and related traits (74). The results indicate that multiple independent factors contribute to yield and biochemical variation in Colocasia. This insight helps in designing a multi-trait selection strategy that can enhance both productivity and quality traits, ensuring superior cultivar development. This statistical approach provides a robust framework for the selection of Colocasia genotypes with optimal agronomic performance, nutritional quality, and biochemical properties, facilitating targeted breeding for improved cultivar development.

## 4 Conclusion

The results of this study indicate significant (*p* < 0.05) variations among the 30 *Colocasia esculenta* genotypes in terms of yield, biochemical composition, mineral content, and antioxidant parameters. Correlation analysis revealed that corm weight and cormel weight can serve as effective selection indices for yield improvement. Notably, Tamachongkham exhibited the highest corm yield, Tamitin had the highest total yield and key minerals (N, K, Zn, Cu, Mn), while Khweng-2 was rich in starch and sugars. Furthermore, Rengama recorded high crude protein and crude fiber, and BCC-2 had superior Fe and Ca + Mg content. The genotypes also demonstrated a diverse nutritional profile, including significant antioxidant properties. Based on these findings, Tamachongkham and Tamitin emerge as the most promising genotypes for future breeding programs aimed at enhancing yield and nutritional quality. These genotypes, along with others exhibiting superior biochemical and mineral traits, should be conserved as a genetic resource for future improvement programs to support food security and sustainability, particularly for the tribal communities of the North-Eastern Hill Region, India. These findings have practical applications in breeding programs for selecting high-yielding and nutrient-rich taro varieties, contributing to food security and crop diversification efforts. Future research should focus on assessing genotype performance under different environmental conditions, exploring anti-nutritional factors and utilizing genomic tools to accelerate the breeding of superior taro cultivars.

## Data Availability

The original contributions presented in the study are included in the article/supplementary material, further inquiries can be directed to the corresponding author.
